# Explainable AI-based feature importance analysis for ovarian cancer classification with ensemble methods

**DOI:** 10.3389/fpubh.2025.1479095

**Published:** 2025-03-26

**Authors:** Ashwini Kodipalli, V. Susheela Devi, Shyamala Guruvare, Taha Ismail

**Affiliations:** ^1^Department of Computer Science and Automation, Indian Institute of Science, Bangalore, Karnataka, India; ^2^School of Computer Science and Engineering, RV University, Bangalore, Karnataka, India; ^3^Department of Obstetrics and Gynecology, Kasturba Medical College, Manipal, Manipal Academy of Higher Education, Manipal, Karnataka, India; ^4^Department of Radiology, Kanachur Institute of Medical Sciences, Mangaluru, Karnataka, India

**Keywords:** interpretable AI, ensemble models, bagging, boosting, machine learning, *p*-value, Cohen’s, SHAP

## Abstract

**Introduction:**

Ovarian Cancer (OC) is one of the leading causes of cancer deaths among women. Despite recent advances in the medical field, such as surgery, chemotherapy, and radiotherapy interventions, there are only marginal improvements in the diagnosis of OC using clinical parameters, as the symptoms are very non-specific at the early stage. Owing to advances in computational algorithms, such as ensemble machine learning, it is now possible to identify complex patterns in clinical parameters. However, these complex patterns do not provide deeper insights into prediction and diagnosis. Explainable artificial intelligence (XAI) models, such as LIME and SHAP Kernels, can provide insights into the decision-making process of ensemble models, thus increasing their applicability.

**Methods:**

The main aim of this study is to design a computer-aided diagnostic system that accurately classifies and detects ovarian cancer. To achieve this objective, a three-stage ensemble model and a game-theoretic approach based on SHAP values were built to evaluate and visualize the results, thus analyzing the important features responsible for prediction.

**Results and Discussion:**

The results demonstrate the efficacy of the proposed model with an accuracy of 98.66%. The proposed model’s consistency and advantages are compared with single classifiers. The SHAP values of the proposed model are validated using conventional statistical methods such as the *p*-test and Cohen’s *d*-test to highlight the efficacy of the proposed method. To further validate the ranking of the features, we compared the *p*-values and Cohen’s *d*-values of the top five and bottom five features. The study proposed and validated an AI-based method for the detection, diagnosis, and prognosis of OC using multi-modal real-life data, which mimics the move of a clinician approach with a demonstration of high performance. The proposed strategy can lead to reliable, accurate, and consistent AI solutions for the detection and management of OC with higher patient experience and outcomes at low cost, low morbidity, and low mortality. This can be beneficial for millions of women living in resource-constrained and challenging economies.

## 1 Introduction

Ovarian cancer (OC) is one of the most fatal gynecological cancers in women. With delayed detection and complexity of disease progression being the main challenges in the treatment of OC, it continues to be a considerable health concern. In 2023, ovarian cancer stands as the most prominent cause of death among gynecological cancers in the United States and is the second most common type of gynecological cancer. Approximately 19,000 new ovarian cancer cases are expected to be diagnosed in the United States annually, with a projected 13,000 casualties (1). Approximately 300,000 new cases are diagnosed annually worldwide (2). Approximately 80% of patients face a recurrence of the disease although they would have undergone initial treatment (3). These facts emphasize the imperative need for developments in early detection and treatment strategies, which is an ongoing research field.

Diagnosis in the initial stages and its precise classification is crucial for cancer diagnosis and the development of personalized treatment plans. Yet, traditional diagnostic methods often fall short of the required accuracy and transparency, restricting their efficacy in clinical practice.

The use of the latest developments in the fields of machine learning (ML) and artificial intelligence (AI) could result in promising improvements in the diagnostic process. These technologies can aid in creating sophisticated models that can analyze large datasets and identify the complex patterns of different cancer types. Ensemble models, which combine the predictive capabilities of multiple classifiers, have exhibited better performance in classification tasks than single classifiers. Regardless of their effectiveness, these models encounter challenges with transparency and interpretability when applied in clinical settings.

The current study intends to focus on these challenges by incorporating an explainable AI (XAI) approach to the interpretation and classification of OC using an ensemble of machine learning models. The key objectives are to utilize the advantages of ensemble learning while applying explainable AI techniques and verify that the model’s decision-making process is transparent and comprehensible to clinicians.

The following are the main contributions to this study:

- A three-stage ensemble model is proposed for accurate classification.
- Explainable AI methods (LIME and SHAP) are applied to gain a deeper understanding of the decision-making process of the proposed model.
- The results of the proposed model are validated using the p-test and Cohen’s *d*-test.
- The feature importance provided by the proposed model is validated using the Wilcoxon signed-rank test (4).

The structure of the paper is organized as follows: Section 2 reviews related studies involving single classifiers, ensemble models, and deep learning approaches. Section 3 details the methodology, including an in-depth description of the datasets and the architectural framework. Section 4 presents the results obtained from the study, and the discussion section provides a detailed comparative study with the existing related studies. Finally, Section 5 concludes with potential directions for future research.

By integrating explainable AI with ensemble learning, this study seeks to bridge the gap between advanced machine learning methodologies and their practical implementation in medical diagnostics. This approach aims to enhance the detection and treatment of ovarian cancer by combining the predictive accuracy of ensemble models with the interpretability provided by explainable AI techniques.

## 2 Related studies

Machine learning (ML) and deep learning (DL) have revolutionized various fields, and their impact on medicine is particularly profound. These technologies leverage vast amounts of data and sophisticated algorithms to enhance diagnostic accuracy, personalize treatment, predict disease outbreaks, and streamline administrative processes. This section provides an overview of how the ML and DL methods have transformed medical practices and research.

### 2.1 Related studies using single classifiers

Nopour et al. (5) used a logistic regression (LR) model and an eXtreme Gradient Boosting (XGBoost) model for OC prediction and reported that XGBoost exhibited better accuracy than the regression model. Li et al. (6) adopted a logistic regression (LR)-based radiomics model out of other omics models (support vector machine (SVM), K-nearest neighbors (KNNs), Random Forest (RF), and XGBoost) and achieved an accuracy of 84.8%. Kori et al. (7) built a model using principal component analysis and ML algorithms that showed 96.7% sensitivity and 100% specificity. Klein et al. (8) built a model using MALDI imaging and ML algorithms such as linear discriminant analysis (LDA), SVM-lin, and SVM-rbf for epithelial ovarian cancer (EOC) histotype classification, yielding a mean accuracy of 80% for LDA, 80% for SVM-lin, and 74% for SVM-rbf. Chao et al. (9) designed an LR model for OC prediction and obtained the area under the curve (AUC), sensitivity, and specificity values of 0.903, 89.2, and 82.3% in the training dataset and 0.891, 88.9, and 76.75% in the test dataset, respectively. Schilling et al. (10) used ML models using K-means clustering, Naive Bayes, logistic regression, and SVM for disease prediction and revealed that the LR model performed the best with a 0.89 f1 score. Hong et al. (11) built a radiomics model based on LASSO Cox regression and reported good discrimination in both training and validation sets with C-indexes of 0.694 (95% confidence interval [CI]: 0.613–0.775) and 0.709 (95% CI: 0.517–0.901), respectively. Bahado-Singh et al. (12) worked with ML algorithms (SVM, LDA, prediction analysis for microarrays (PAMs), and generalized linear model (GLM)) and obtained optimal results of AUC value (95% CI) 1.00 (0.9000–1.0) with SVM and AUC (95% CI) 0.99 (0.9000–1.0) with GLM, PAMs, and LDA, along with achieving an AUC value (95% CI) 1.00 (0.9–1.0) with SVM models for CpG analysis. Sheela Lavanya et al. (13) applied ML techniques (KNNs, SVM, DTs) alongside explainable AI to aid the early detection of OC and demonstrated that SVM performed with 85% base model accuracy. Feng Zhan et al. (14) designed the LR radiomic model to predict lymphocyte-specific protein tyrosine kinase (LCK) expression and overall survival in high-grade serous ovarian cancer (HGSOC) patients and achieved AUC values of 0.879 and 0.834, respectively. A.D Coles et al. (15) performed a comparative analysis of the RF and LR models for retrospective detection of OC recurrences from chemotherapy data and revealed that RF achieved the highest F1 score among the two. Alexander Laios et al. (16) performed ML-based risk prediction using the KNN and LDA techniques and QDA algorithms that achieved predictive accuracies of 0.80, 0.90, and 0.92, respectively.

### 2.2 Related studies based on ensemble models

Subsequent studies used the following ensemble models for the prediction of OC intrinsically: Random Forest (RF), XGBoost, gradient-boosted trees (GBT), and Bagging. Gong et al. (17) used nine supervised ML classifiers including RF and GBT, out of which RF performed with high stability and an accuracy of 0.60 including gut microbiota for chemoresistance to OC prediction using the RF model with an AUC value of 0.909. Ahamad et al. (18) utilized a combination of XGBoost, GBT, and light gradient boosting machine (LGBM) to build classifier models, and the predictive analysis results revealed an accuracy of 91%. Hamidi et al. (19) developed Boruta, a novel RF feature selection-based ML model, for identifying important biomarkers. Zhao et al. (20) used LASSO regression, ridge regression, XGBoost, RF, and AdaBoost to build a LASSO Cox regression model. By performing decision curve analysis, the resulting nomogram indicated that the combined model for predicting 1-year and 3-year survival probabilities provided an optimal net benefit compared to using a single indicator. Wadapurkar et al. (21) applied next-generation sequencing analysis to cancer driver genes and OC prediction using the XGBoost classification model and yielded an accuracy of 0.946. Abuzinadah et al. (22) used the RF model, GBM, and also ensemble GBM + XGB models. GBM was reported to be the best with an accuracy of 87.14%, a recall of 87.53%, and a precision of 87.58%. Zeng et al. (23) used a new multi-omics model including all the features and showed the best prediction performance with an AUC value of 0.911. Piedimonte et al. (24) used a Random Forest model that was trained to predict the dichotomous outcome of optimal cytoreduction and obtained an AUC of 99.8%. Cheng et al. (25) used a gradient-boosting decision tree algorithm in which the AUC value of the signature gene pair was 0.9658, whereas the AUC value of the individual signature gene-based prediction was only 0.6823. Seri Jeong et al. (26) utilized the RF model for differentiating benign and malignant OC using combined cancer markers and achieved receiver operating characteristic (ROC) AUC values of 0.707, 0.680, 0.643, 0.657, and 0.624 for the biomarkers ROMA, HE4, CA125, LD, and NLR, respectively. Maria et al. (27) used an XAI-based ensemble model for the accurate classification of ovarian cancer and achieved an accuracy of 83.2%. Additionally, the model proved to be highly effective in handling imbalanced datasets. Annarita et al. (28) proposed a waterfall-based classification model for the classification of clinical data and ultrasound indicators and achieved an accuracy of 86.36%.

### 2.3 Related studies based on deep learning models

Wu et al. (29) used an attention-based network by using a pre-trained model ResNet50 on ImageNet to obtain a C-index value of 0.5789 (0.5096–0.6053) and a p-value of 0.00845, while the risk score indicated good prediction ability in the homologous recombination deficiency (HRD+) subgroup. Feng et al. (30) used a backpropagation neural network model by using a pre-trained ResNet50 which outperformed the U-net-based network architecture by obtaining 89.11% average sensitivity and 96.37% specificity. Suganya et al. (31) used MALDI imaging for the EOC classification. Out of several ML and DL models used in the research, neural network and convolutional neural network (CNN) were most suitable for EOC classification with an accuracy of 83% for NN, 85% for CNN, and a sensitivity of 69–100% as well as specificity of 90–99% for both the models, respectively. Rahul Mishra et al. (32) used the Convolutional Neural Networks with Gray Wolf Optimization (CNNGWO) model and reported an improved diagnostic accuracy of OC to 98%, while Talib et al. (33) trained a simple CNN-based DL model on 1798 images and achieved an accuracy of 81% and an AUC value of 0.89. Kasture et al. (34) used outclassed traditional CNN algorithms, deep CNN, and pre-trained AlexNet models, improving the accuracy from 70 to 89.93%. Reilly et al. (35) proposed an MIA3G model (deep feed-forward neural network) for OC risk assessment and achieved a sensitivity of 89.8%, specificity of 84.02%, positive predictive value (PPV) of 22.455, and negative predictive value (NPV) of 99.38%. David Joon Ho et al. (36) worked with a deep multi-magnification network model (DMMN) for OC segmentation and obtained 86% recall and 84% precision. Ziyambe et al. (37) built a CNN architecture model using the Xception network and obtained 94.43% accuracy, 95.02% sensitivity, and 93.16% specificity, making it a potential tool in assisting physicians for predicting OC. Dingdu Hu et al. (38) conducted automatic segmentations of T2-weighted MRI using DL algorithms (CNN, U-Net, DeepLabv3, U-Net++, and PSPNet) along with transformers (TransUnet and Swin-Unet), and reported that U-Net++ performed the best with precision and recall rates of 83.8 and 88.2% in the internal test set and 82.5 and 72.5% in the external test set, respectively. Ravishankar et al. (39) proposed a fuzzy CNN-based classifier with an initial accuracy of 98.37%, which later achieved 100% accuracy occasionally with an increase in several epochs. Wang et al. (40) used two improved DL-based methods for predicting bevacizumab efficacy in EOC patients based on histopathological images and revealed that the proposed model using the MSH2 protein expression performed excellently with 100% accuracy, sensitivity, and specificity, respectively. Ju Young et al. (41) developed a U-Net-based DL algorithm for the characterization of cancer-associated thrombosis of OC and achieved outstanding results with an AUC value of 0.99 along with high sensitivity and specificity. Jeya Sundari et al. (42) developed an intelligent black widow optimization on image enhancement with a DL-based ovarian tumor diagnosis model, which achieved a maximum contrast of 0.97, a contrast-to-noise ratio (CNR) of 92.74%, a weighted peak signal-to-noise ratio (WPSNR) of 20.43, and a homogeneity of 0.94. Rui Yin et al. (43) worked with a multitask deep learning model for high-grade serous ovarian carcinoma (HGSOC) prediction using CT images of OC, which resulted in AUC values of 0.87 (0.83–0.89), 0.88 (0.86–0.91), 0.86 (0.82–0.89), and 0.79 (0.75–0.82) in the training sets, validation sets, prospective sets, and external sets, respectively. Yang et al. (44) developed the Ovarian Cancer Digital Pathology Index (OCDPI), a graph-based DL model, for prediction and treatment response in OC patients using histopathological images and demonstrated prognostic ability for overall survival prediction in the prostate, lung, colorectal, and ovarian cancer (PLCO; HR, 1.916; 95% CI, 1.380–2.660; log-rank test, *p* < 0.001) and Harbin Medical University Cancer hospital (HMUCH) (HR, 2.796; 95% CI, 1.404–5.568; log-rank test, *p* = 0.0022) cohorts. It was observed that the performance of OCDPI was good in patients with low-grade tumors or fresh frozen slides. Laios et al. (45) developed a predictive model using XGBoost and the deep neural network for the prediction of epithelial ovarian cancer and used a SHAP technique to discuss how intraoperative decisions during EOC cytoreduction can integrate human factors along with factual knowledge to optimize the selected trade-off in surgical effort. Huang et al. (4) emphasized classification tasks and used a game-theoretic framework using Shap values for model development, evaluation, and result visualization. The case study findings highlighted the effectiveness, reliability, and benefits of the ML pipeline in comparison to traditional statistical methods.

## 3 Methodology

### 3.1 Data collection and description

The dataset comprises 1,500 cases of ovarian tumors, classified into borderline, malignant, and benign types. The authors meticulously transferred the data manually from medical records to an Excel file, following ethical medical practices under the vigilance of medical practitioners at the Kasturba Medical College, Karnataka, India. Each row represents a unique patient and the columns capture various clinical features pertinent to the presence or absence of ovarian cancer. The raw data from the records were structured into the following 28 columns in the Excel file: clinical parameters such as age, parity, family history, oral contraception, age at menarche, menopause, abdominal pain, menstrual abnormalities, dysmenorrhea, abdominal distention, loss of appetite, edema, pallor, P/V tenderness, and abdominal mass; ultrasound or CT parameters: bilateral, ascites, solid area, papillary projection, thick septa, vascularity, contrast enhancement, lymph nodes, and deposits; and tumor markers such as CA125, CA19-9, and CEA and the corresponding category or class label. There are three classes: 0 – benign, 1 – border, and 2 – malignant.

Some of the feature descriptions are as follows:

- Parity: number of full-term pregnancies.
- Oral contraception: history of using oral contraceptive pills.
- Dysmenorrhea: painful menstruation.
- Abdominal distention: swelling or enlargement of the abdomen.
- Edema: swelling caused by excess fluid trapped in body tissues.
- Nodes: presence of swollen lymph nodes.
- Pallor: unusual paleness of the skin.
- P/V tenderness: pain during a pelvic examination.
- Abdominal mass: detectable lump in the abdomen.
- Bilateral: side of the body where the tumor is located.
- Ascites: accumulation of fluid in the peritoneal cavity.
- Solid area: presence of solid regions within the tumor.
- Papillary projection: finger-like extensions seen in tumors.
- Thick septa: thickened partitions within the tumor.
- Vascularity: blood supply to the tumor.
- Contrast enhancement: increase in the visibility of structures using contrast medium in imaging.
- Deposits: tumor spread or deposits in the abdomen.
- CA125: blood marker often elevated in ovarian cancer.
- CA19-9: tumor marker used in the diagnosis of ovarian cancer.
- CEA: carcinoembryonic antigen, another tumor marker.

This dataset served as a vital tool for studying the clinical features and diagnostic markers of ovarian tumors, aiding in advancing medical research and treatment strategies.

### 3.2 Architectural framework

**Figure 1:**
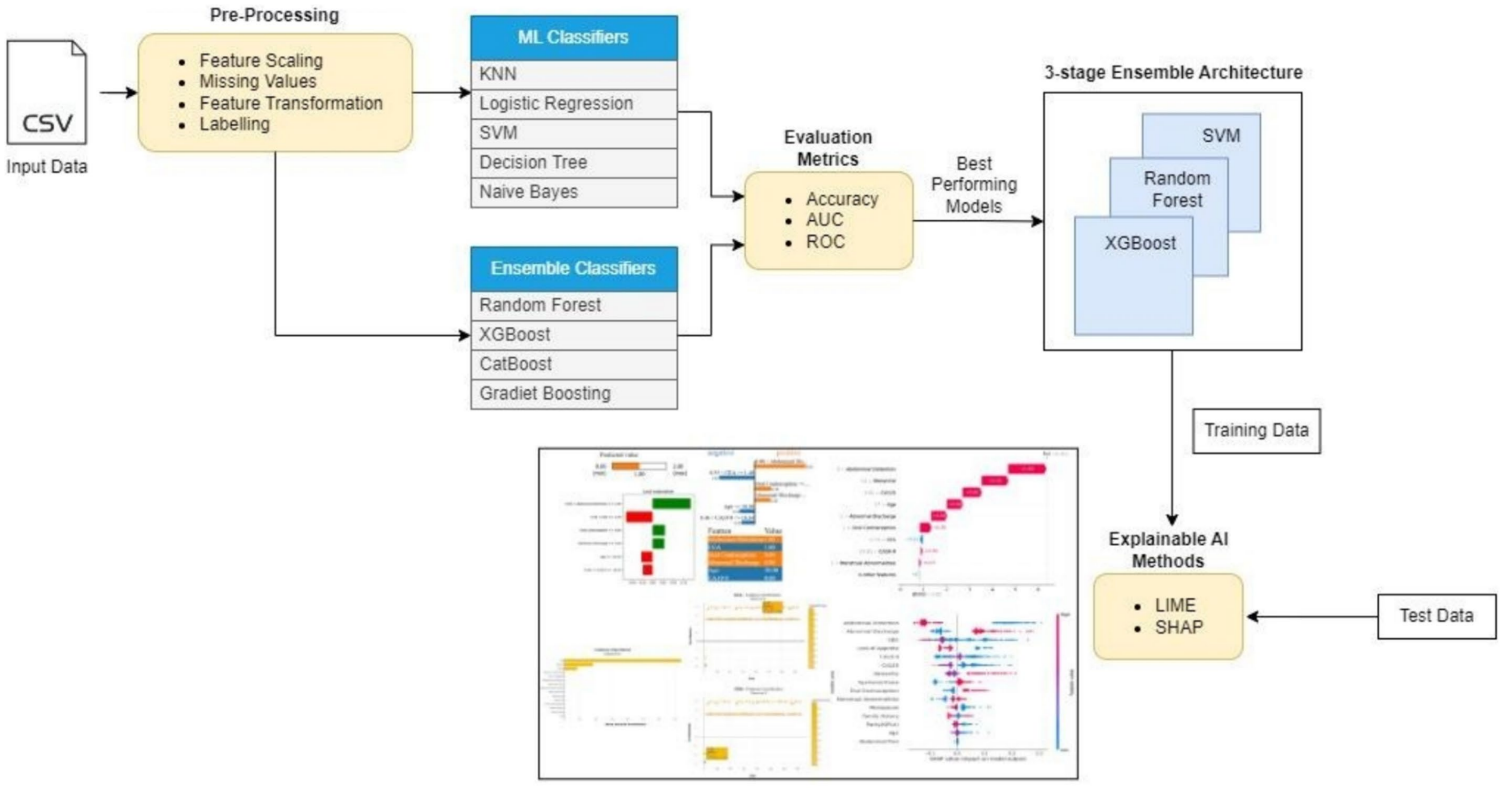
Process diagram.

Figure 1 depicts the process flow followed in the current study. The dataset consists of 1,500 samples, with benign, malignant, and border cases comprising 500 samples each. The data are stored in a CSV file. This is considered the Input Data file that holds the raw data. Further steps were performed on this data. Preprocessing steps such as feature scaling were performed using a standard min–max scaler. A standard min–max scaler was applied to rescale all features to a uniform range, typically [0, 1]. This ensured that features with different scales did not disproportionately influence the model. Feature Transformation using exponential transformation was used to address skewed data distributions; the model was enhanced to capture patterns in the data along with labeling instances into correct categories, and missing values were imputed using mean imputation, replacing missing entries with the mean of the respective features. This approach preserved the overall distribution of the data and maintained dataset integrity without introducing significant bias. The processed data were then provided as input for several machine learning classifier models. The ML classifiers K-nearest neighbors (KNNs), logistic regression (LR), support vector machine (SVM), decision trees (DTs), and Naive Bayes (NB) were used in the current study. For the ensemble classifiers, models such as Random Forest (RF), XGBoost, CatBoost, and GradientBoost were applied. All classifiers were evaluated based on their performance using evaluation metrics such as accuracy, AUC, and ROC. The best-performing models among all the models were chosen. Random Forest, XGBoost, and SVM with accuracies of 98.13, 97, and 97.06%, respectively, were the top three best-performing models and were used to design a three-stage ensemble architecture. These scores represent the performance of the models on the test data. Explainable AI methods were applied to the test data results and passed onto the final ensemble model. LIME and SHAP were used to visualize and explain the predictions made by the model, as they are widely used for explanation across domains.

## 4 Results and discussion

### 4.1 Statistical analysis using machine learning models

**Figure 2:**
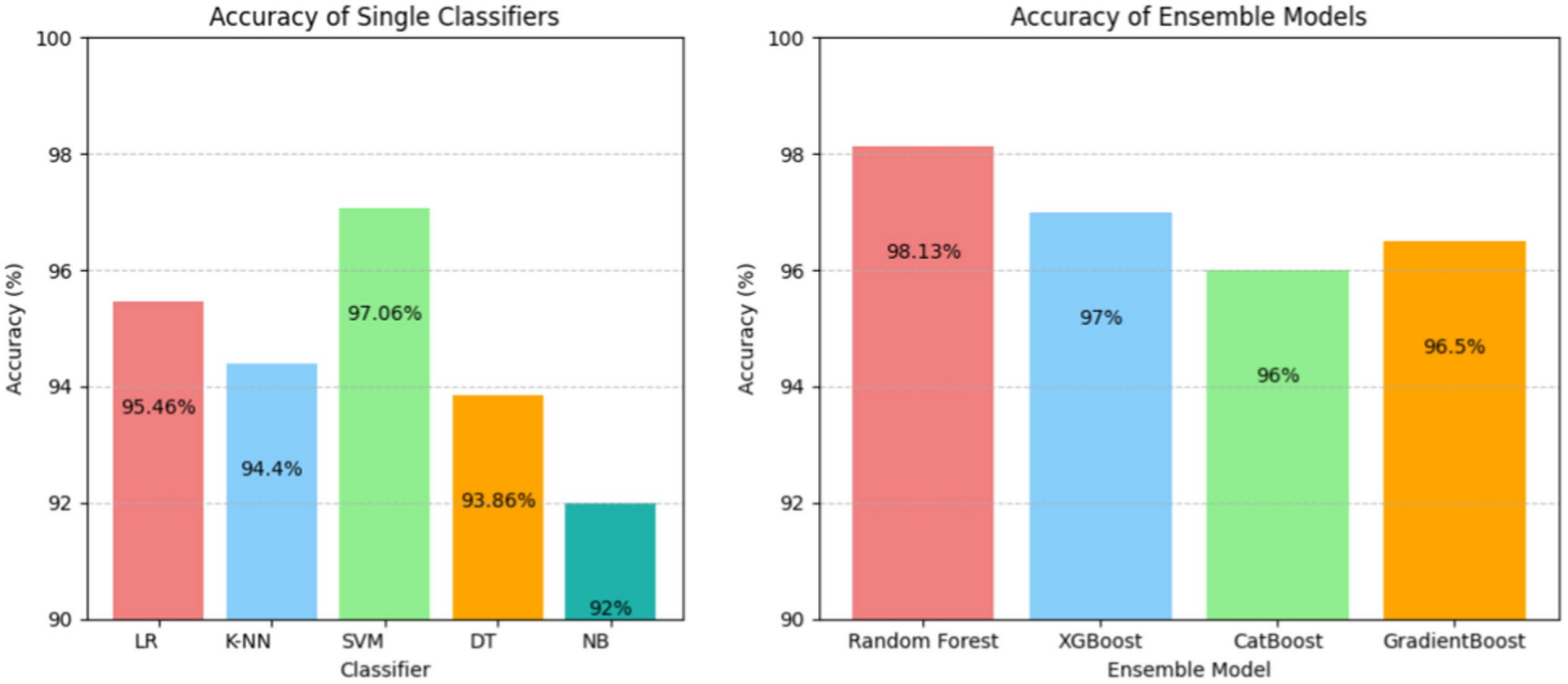
Accuracy of the single classifier (left) and ensemble classifiers (right).

**Figure 3:**
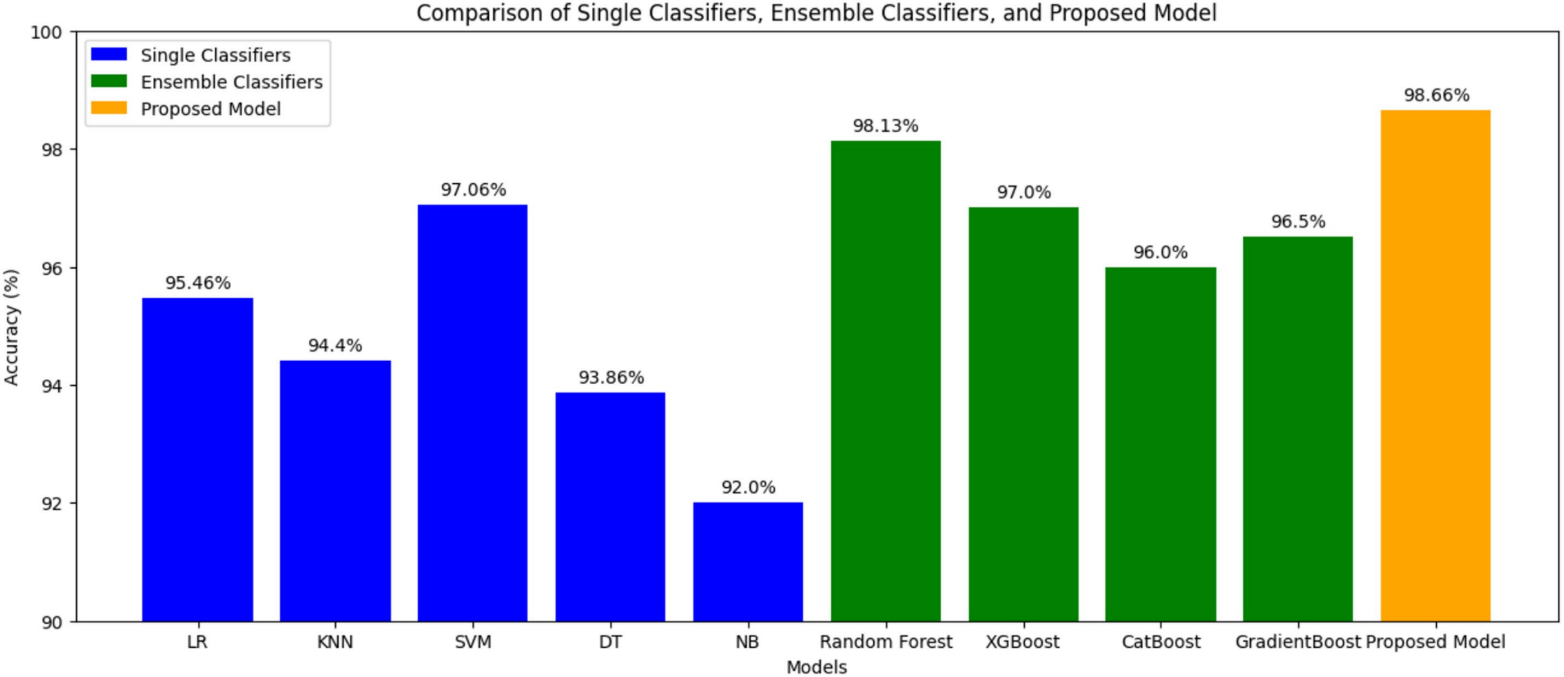
Comparison of single, ensemble, and three-stage ensemble models.

The results have been computed by considering the complete dataset of clinical parameters, tumor markers, and ultrasound parameters. The accuracy rates of single classifiers and ensemble classifiers are depicted as bar charts in Figure 2, respectively. It can be observed that SVM and Random Forest exhibit the highest accuracy among the single classifiers and the ensemble models, respectively. Only SVM, Random Forest (RF), and XGBoost have an accuracy of 97% or above, with 97.06, 98.13, and 97%, respectively. Therefore, these models were used to build the three-stage ensemble classifier. In Figure 3, the accuracy rates of all the models used in the current study have been plotted along with the proposed three-stage ensemble classifier.

**Figure 4:**
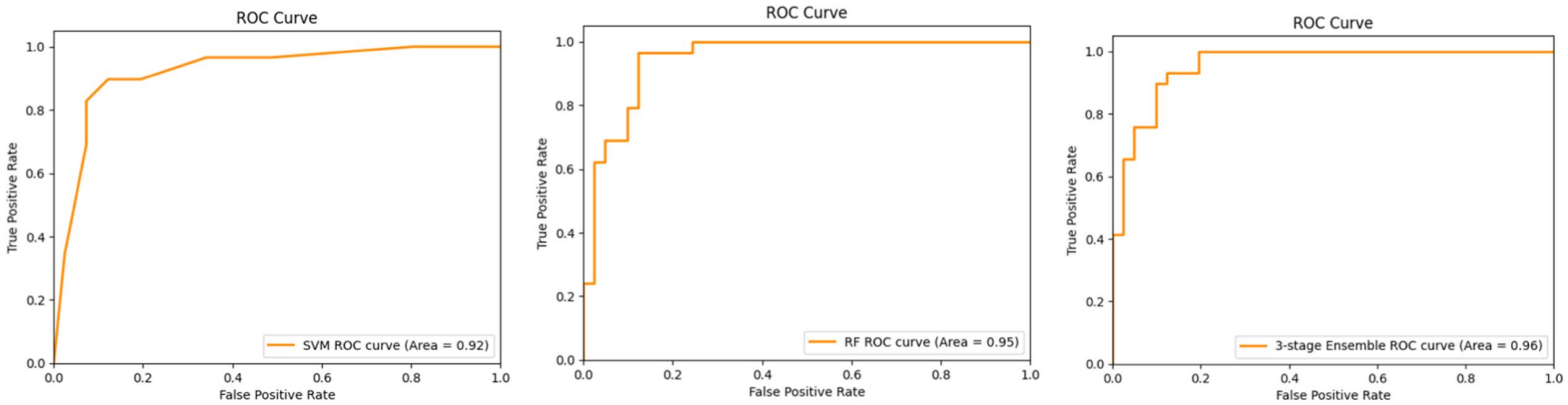
ROC curves for the SVM (left), RF (middle), and three-stage ensemble architecture (right).

**Figure 5:**
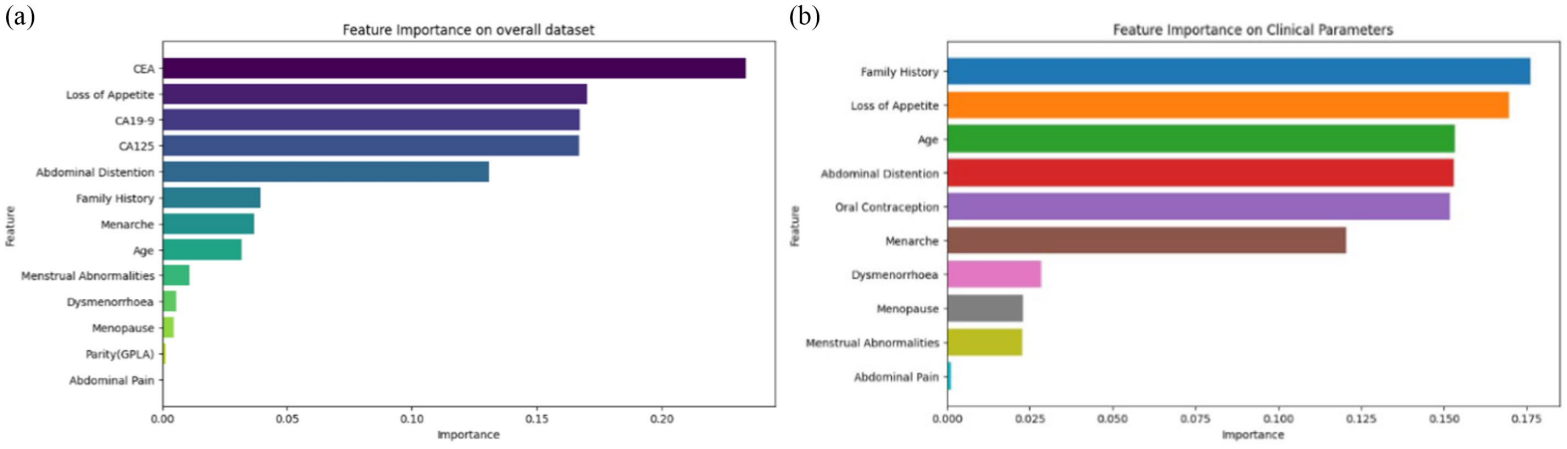
Feature importance **(A)** on the overall dataset and **(B)** only on clinical parameters.

Figure 4 shows the ROC curves for SVM, Random Forest, and the proposed three-stage ensemble models. Their AUC–ROC values are 0.92, 0.95, and 0.96, respectively. It is evident from the figure that the proposed model exhibits the best predictive behavior. In machine learning, feature importance is a technique that assigns a score to input features based on their importance for predicting the target variables. Since the proposed model is a combination of Random Forest, SVM, and XGBoost, it naturally provides the feature importance scores as they are the tree-based model. The RF model gives the feature importance based on the entropy score for the classification tree and aggregates the scores of each feature across all the trees in the forest. Each tree’s feature importance is calculated based on the reduction in impurity (entropy value). Similar to RF, GradientBoost calculates the feature importance score using the total reduction in the log loss. The feature importance predicted on the overall dataset and only on the clinical parameters is shown in Figures 5A,B, respectively.

Figure 5A shows the feature importance of each feature contributing to ovarian malignancy. It is evident that CEA, loss of appetite, CA19-9, CA125, abdominal distention, family history, age of menarche, and age are the highest contributors and more prominently influence the decision of which category the particular case will fall into. It can be seen from Figure 5B that the top features family history, loss of appetite, age, abdominal distention, oral contraception, and age of menarche are the highest contributors among clinical parameters and more prominently influence the decision of which category the particular case will fall into. In contrast, the features such as dysmenorrhea, menopause, menstrual abnormalities, and abdominal pain are the lowest contributors clinically, however, but are of significance when accounted for as an isolated event.

### 4.2 Analysis using explainable AI

#### 4.2.1 Lime

**Figure 6:**
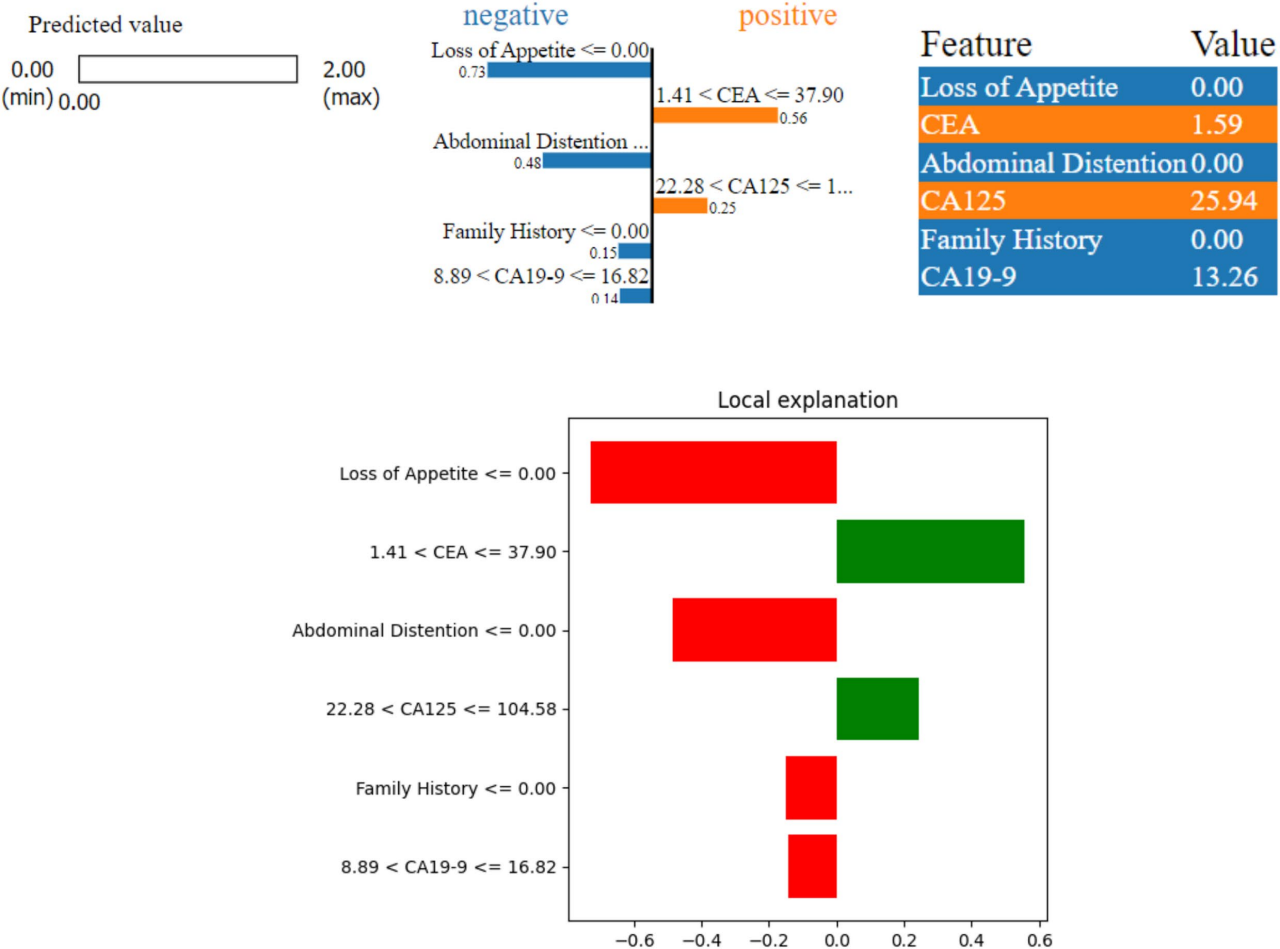
LIME instance for Class 0 (benign).

LIME helps in understanding how specific features of patients’ data influence the model prediction. As shown in Figure 6, the model predicted that the patients do not have a serious condition (benign). Out of the six features considered, ‘Abdominal Distention’, ‘Loss of Appetite’, ‘Family History’, and ‘CA19-9′ indicate that there is no serious condition (negative contribution). On the other hand, ‘CEA’ and ‘CA125’ indicate slight risk (positive contribution) at the very initial stages. The actual values for these features are as follows: ‘Abdominal Distention’ at 0.0, ‘CEA’ at 1.59, ‘CA19-9′ at 13.26, ‘Loss of Appetite’ at 0.0, ‘Family History’ at 0.0, and ‘CA125’ at 25.94. Because ‘Loss of Appetite’, ‘Abdominal Distention’, ‘Family History’, and ‘CA19-9′ fall within safe ranges, they contribute to the model prediction as benign. Although CEA and CA125 values are positively contributing, the values are very negligible. CA125 value is 25.94, which is outside the safe range, but the other factors are negatively contributing, and thus the case can be considered benign.

**Figure 7:**
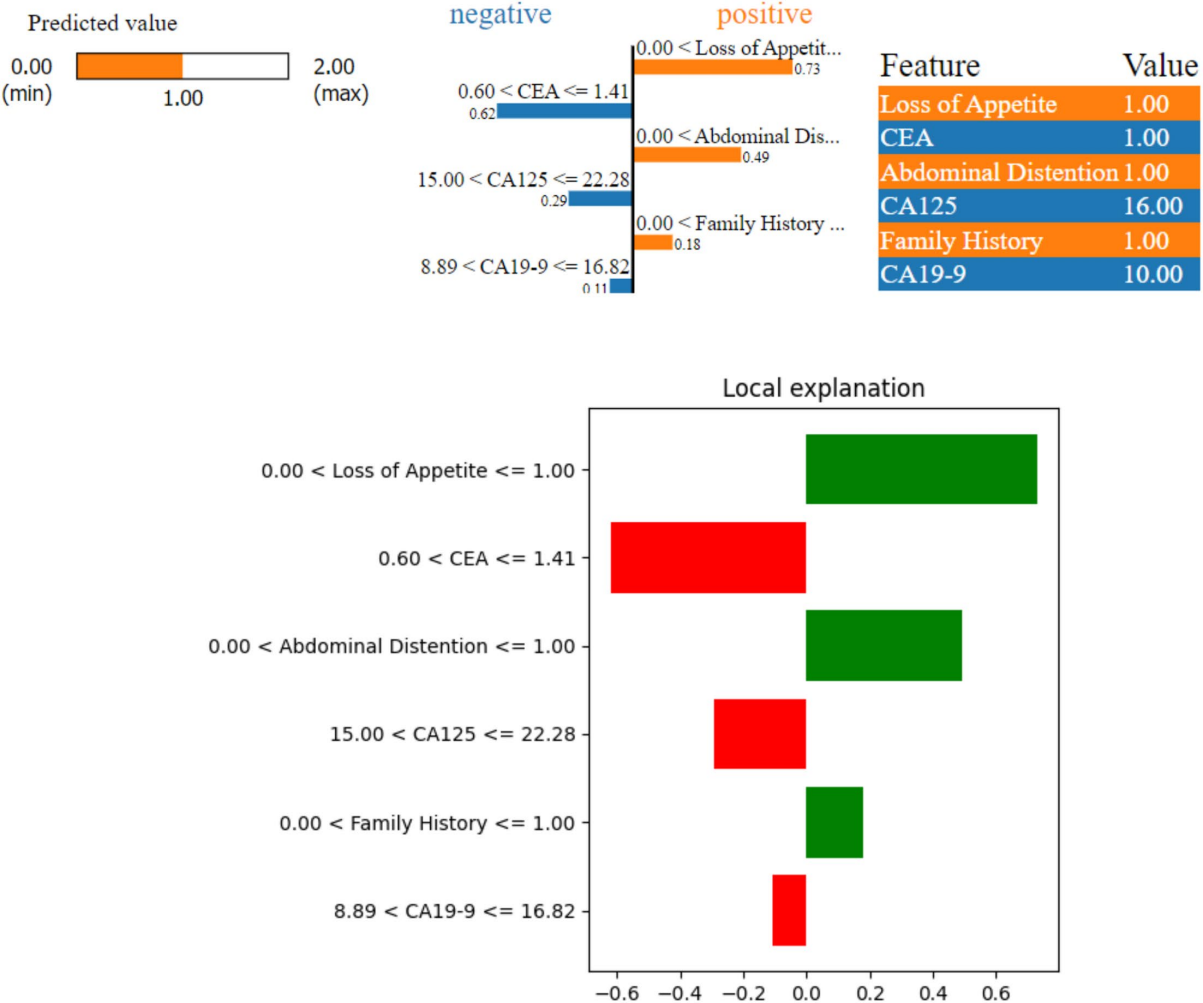
LIME instance for Class 1 (border case).

Figure 7 shows a borderline case where the model predicted a possible risk (Class 1) of ovarian malignancy. Here, three features—‘Abdominal Distention’, ‘Loss of Appetite’, and ‘Family History’—indicate a higher risk (positive contribution), while ‘CEA’, ‘CA19-9’, and ‘CA125’ suggest a lower risk (negative contribution) as they are not de-ranged values. The values of these features are as follows: ‘Abdominal Distention’ at 1.0, ‘Loss of Appetite’ at 1.0, ‘CA19-9’ at 10.0, ‘CEA’ at 1.0, and ‘CA125’ at 16.0. Because ‘Abdominal Distention’, ‘Loss of Appetite’, and ‘Family History’ are within the concerning ranges, they contribute to the model prediction of possible risk, with the border values of CEA being yes and CA125 toward the border value.

**Figure 8:**
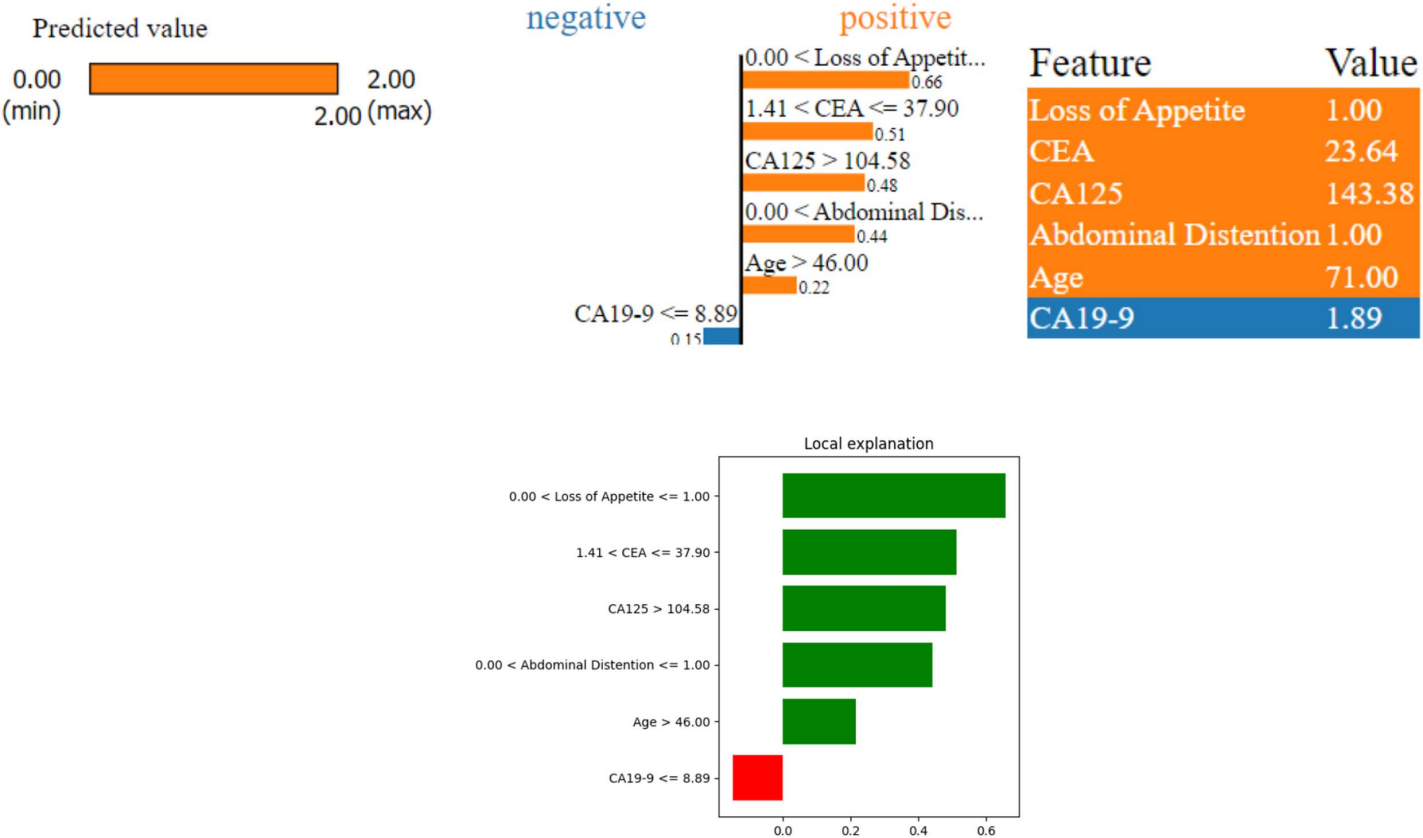
LIME instance for Class 2 (malignant case).

Figure 8 shows that the model predicted a serious ovarian condition (malignant). The features—‘Loss of Appetite’, ‘CEA’, ‘CA125’, ‘Abdominal Distention’, and ‘Age’—indicate a higher risk (positive contribution). The values of these features are as follows: ‘Loss of Appetite’ at 1.00 indicates that the person is having a loss of appetite issue, ‘Abdominal Distention’ at 1.0, ‘CEA’ at 23.64 is also on the higher side, ‘CA125’ at 143.38, ‘Age’ at 71, and ‘CA19-9’ at 1.89. Since all the features except CA19-9 contribute positively, the model prediction is revealed as malignant. The important factor to be considered in this case is the age which is 71; the values of other factors such as CA125, loss of appetite, and abdominal distention are high, leading to malignant prediction.

#### 4.2.2 SHAP

**Figure 9:**
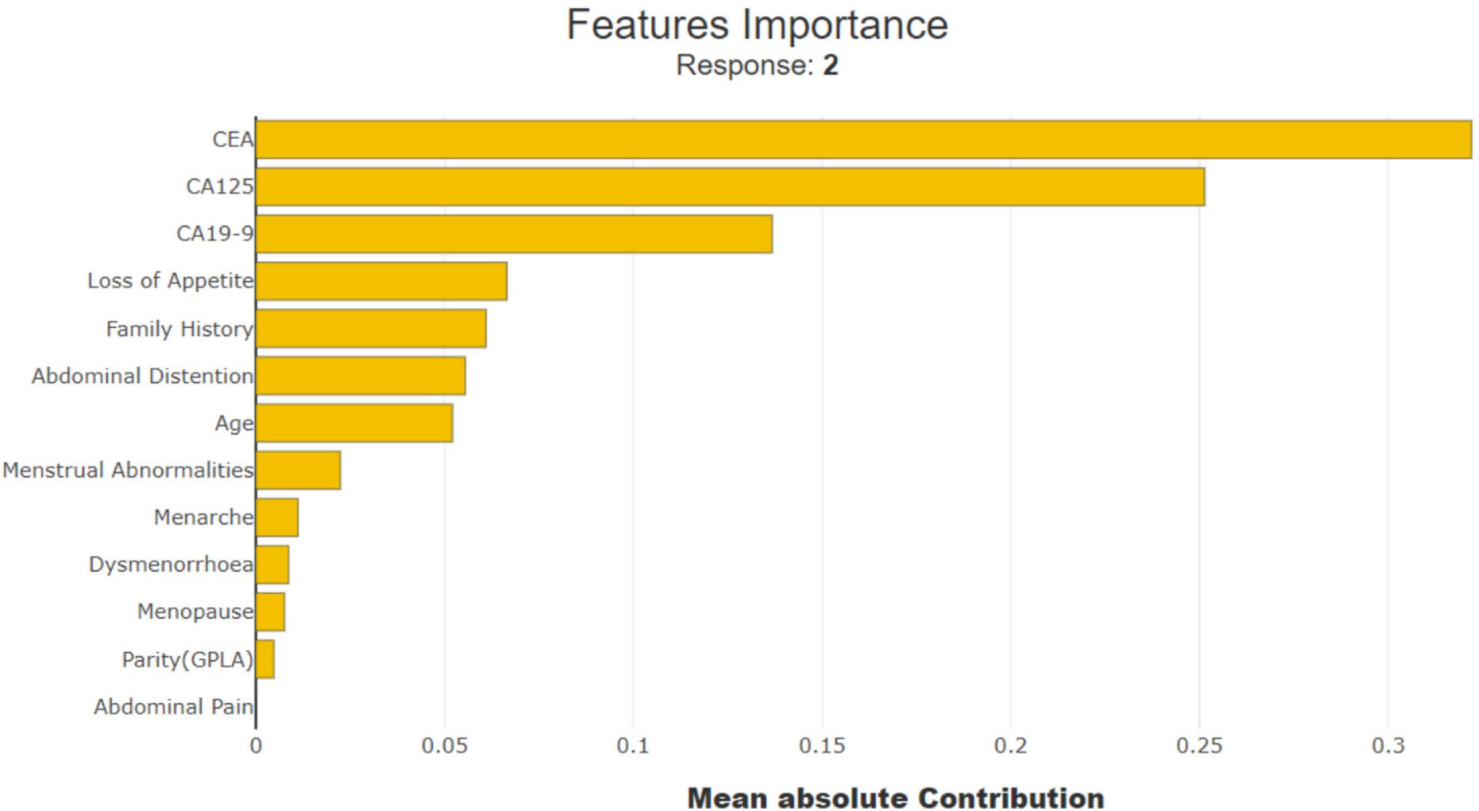
Importance of SHAP feature.

SHAP values help in understanding the importance of each feature in the model prediction for ovarian lesions. As shown in Figure 9, ‘CEA’, ‘CA19-9’, ‘CA125’, ‘Loss of Appetite’, ‘Family History’, and ‘Abdominal Distention’ are the most important features in determining the model predictions; LIME also used the same features for predicting the cases. This indicates that these six features have the most significant impact on the model predicting a benign, borderline, or malignant condition.

**Figure 10:**
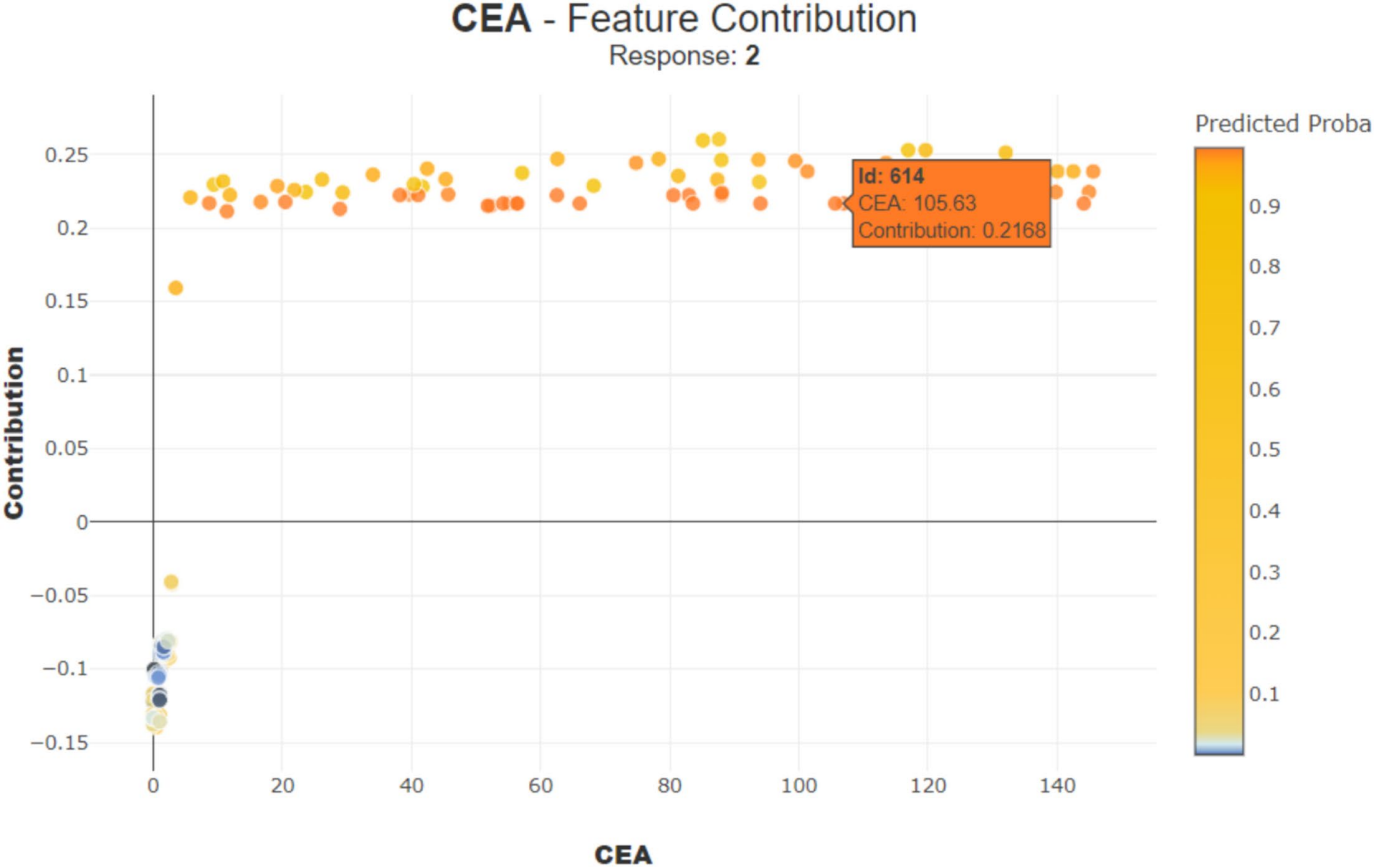
Contribution of CEA for observation number 614.

Figure 10 shows how the feature ‘CEA (Carcinoembryonic Antigen)’ influences the prediction of a specific patient (observation number 9614). With a ‘CEA’ value of 105.63, it contributes positively by 0.2168 points, indicating a higher risk.

**Figure 11:**
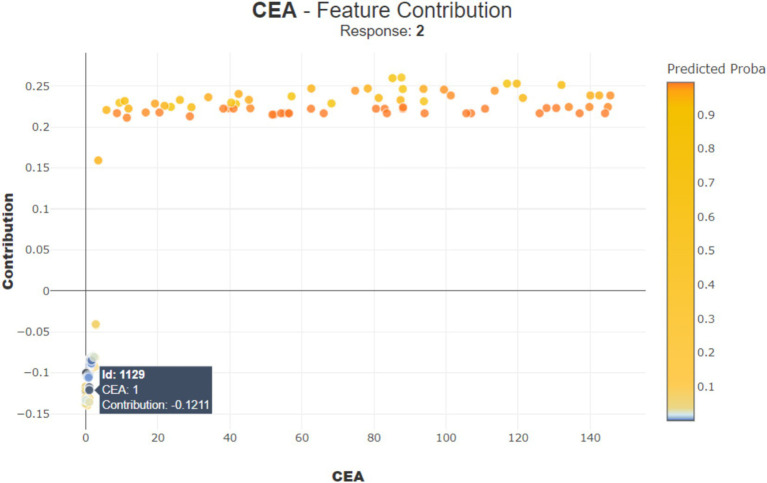
Contribution of CEA for observation number 1129.

In Figure 11, for another patient (observation number 892), the ‘CEA’ value is 1, contributing negatively by −0.1211 points, suggesting a lower risk. These examples show that the impact of each feature can be positive or negative depending on its value, helping doctors understand how different aspects of patient data influence the model prediction.

##### 4.2.2.1 Tree SHAP

Interpreting a stacked bar plot of Tree SHAP values with mean SHAP values involves an understanding of how each feature contributes to the model’s predictions on average. This helps in identifying which features are the most influential and how they collectively affect the model output relative to the average prediction.

**Figure 12:**
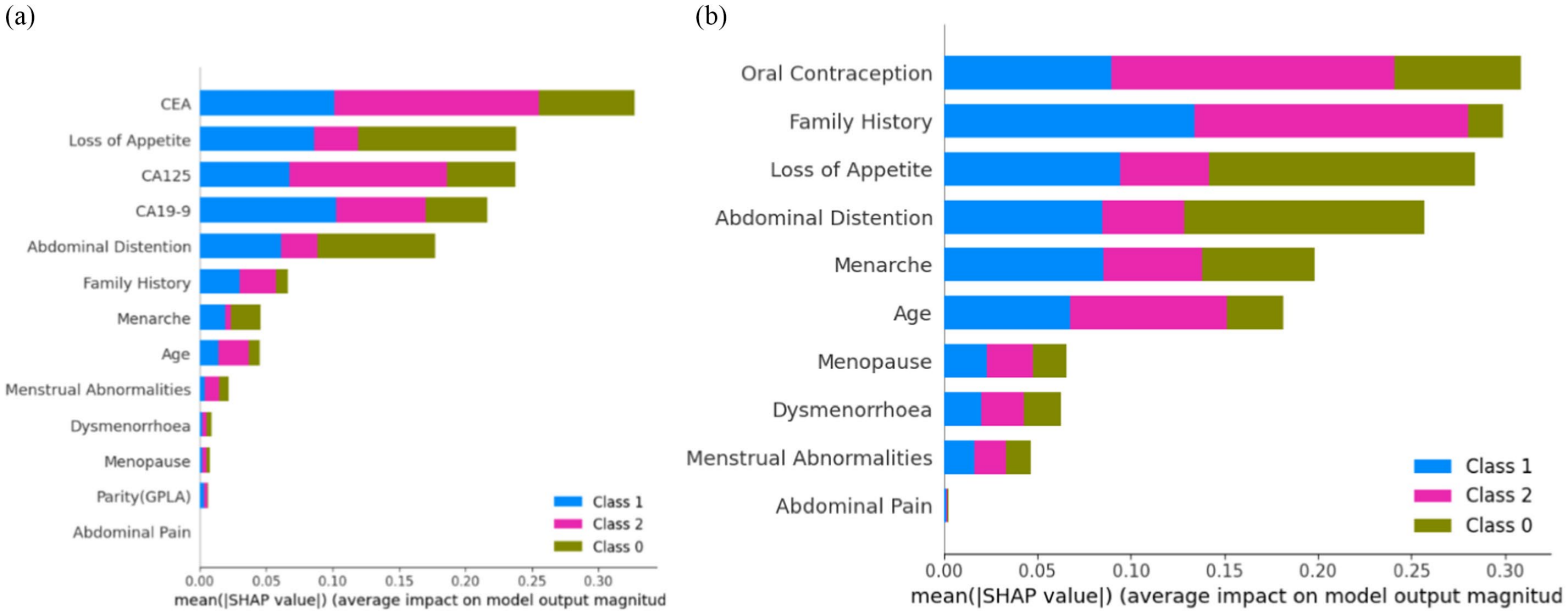
Feature importance **(A)** on the overall dataset and **(B)** on clinical parameters.

Figure 12A shows that ‘CEA’, ‘Loss of Appetite’, ‘CA125’, ‘CA19-9’, ‘Abdominal Distention’, ‘Family History’, ‘Menarche’, ‘Age’, and ‘Menstrual Abnormalities’ are the top nine features attributing to model prediction in cases of ovarian lesions according to the Tree SHAP Explainer. The bar against ‘CEA’ shows that this feature is highly influential in deciding ‘Class 2’ when compared to ‘Class 1’ and ‘Class 0’. The bar against ‘Loss of Appetite’ shows that this feature is highly influential in deciding ‘Class 0’ and ‘Class 1’ when compared to ‘Class 2’. The bar against ‘CA125’ shows that this feature is highly important for predicting ‘Class 2’ when compared to the other two classes. The bar against ‘Abdominal Distention’ shows that this feature is important for instances that belong to ‘Class 0’. Figure 12B shows that ‘Oral Contraception’, ‘Family History’, ‘Loss of Appetite’, ‘Abdominal Distention’, and ‘Menarche’ are the top five features contributing clinically to the model prediction according to the Tree SHAP explainer. The bar against ‘Family History’ shows that this feature is highly influential in deciding Classes 1 and 2 when compared to Class 0. The bar against ‘Age’ shows that this feature is highly influential in deciding Class 2 and Class 1 when compared with Class 0. The bar against ‘Oral Contraception’ is highly influential in deciding Class 2 when compared to Classes 1 and 0.

From the clinician’s point of view, these observations indicate the weightage of these parameters as the conditions for prediction and suggest positive and negative predictive roles for each of the contributors. CEA has the highest positive predictive value for malignancy. CA19-9 has the highest predictive value for borderline tumors, whereas CA125, being a non-specific marker, is not as specifically predictive as any of the categories. For example, if SHAP predicts a tumor as malignant based on CEA and the same mass as benign based on CA125, it is more likely a malignant mass because CEA as an independent factor has the highest reliable predictivity. In the prediction model that included clinical and tumor marker parameters, abdominal distension had more of a negative predictive value than other parameters, indicating that the absence of abdominal distension rules out malignancy and the mass is likely to be benign. However, when only clinical parameters were considered in the prediction model and tumor markers were not included, abdominal distension had the predictive capacity to indicate malignancy by its presence.

##### 4.2.2.2 Sampling SHAP

Interpreting a stacked bar plot of Sampling SHAP values with mean SHAP values involves an understanding of how each feature contributes to the model predictions, considering the variability introduced by the sampling process. This approach helps in identifying influential features and their average impact on model predictions across different samples.

**Figure 13:**
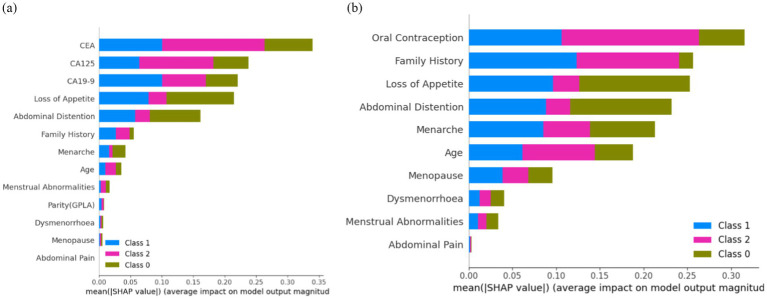
Feature importance **(A)** on the overall dataset and **(B)** on clinical parameters.

Figure 13A shows that ‘CEA’, ‘CA125’, ‘CA19-9’, ‘Loss of Appetite’, ‘Abdominal Distention’, ‘Family History’, ‘Menarche’, ‘Age’, and ‘Menstrual Abnormalities’ are the top nine features contributing to the model prediction of ovarian lesions according to the Sampling SHAP Explainer. The bar against ‘CEA’ shows that this feature is highly influential in deciding ‘Class 2’ when compared to ‘Class 0’ and ‘Class 1’. The bar against ‘CA125’ is highly influential in deciding Class 2 when compared to ‘Class 0’ and ‘Class 1’. The bar against ‘CA19-9’ shows that this feature is highly important for predicting ‘Class 1’ when compared to the other two classes. The bar against ‘Abdominal Distention’ shows that this feature is important for instances that belong to ‘Class 0’. Sampling SHAP was used when exact computation (as done in Tree SHAP) was impractical due to computational constraints. It involved approximating SHAP values through sampling subsets of features or instances. Figure 13B shows that ‘Oral Contraception, ‘Family History’, ‘Loss of Appetite’, ‘Abdominal Distention’, and ‘Menarche’ are the top five features contributing clinically to model prediction as per the Sampling SHAP Explainer. The bar against the features oral contraception, age, and family history shows that these features are highly influential in deciding ‘Class 1 and 2′ when compared to ‘Class 0′. The bar against the features ‘Loss of Appetite’ and ‘Abdominal Distention’ is highly influential in deciding ‘Class 0 and 1′ when compared to ‘Class 2′.

##### 4.2.2.3 Kernel SHAP

Interpreting a stacked bar plot of Kernel SHAP values with mean SHAP values involves an understanding of how each feature contributes to the model prediction, considering the kernel-based approach used for SHAP value computation. This approach helps in identifying influential features and their average impact on model predictions across different instances or data subsets.

**Figure 14:**
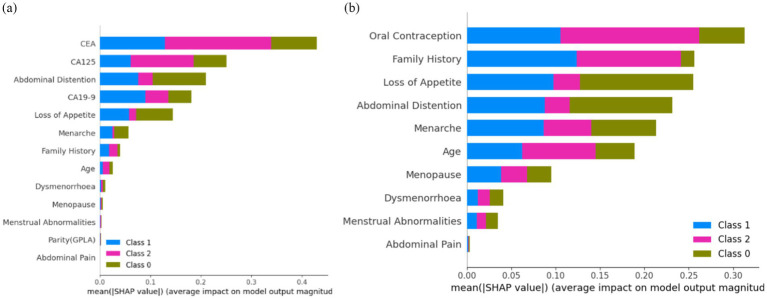
Feature importance **(A)** on the overall dataset and **(B)** on clinical parameters.

Figure 14A shows that ‘CEA’, ‘CA125’, ‘Abdominal Distention’, ‘CA19-9’, ‘Loss of Appetite’, ‘Menarche’, ‘Family History’, ‘Age’, and ‘Dysmenorrhoea’ are the top nine features contributing to the model prediction of ovarian lesions according to the Kernel SHAP Explainer. The bar against ‘CEA’ is highly influential in deciding between ‘Class 2’ and ‘Class 1’ when compared to ‘Class 0’. The bar against ‘CA19-9’ shows that this is highly important for predicting ‘Class 1’ when compared to the other two classes. The bar against ‘CA125’ is highly influential in deciding ‘Class 2’ when compared to ‘Class 0 and Class 2’. The bar against ‘Abdominal Distention’ shows that this feature is important for instances that belong to ‘Class 0’. Sampling SHAP was used when exact computation (as conducted in Tree SHAP) was impractical due to computational constraints. This involved approximating SHAP values through sampling subsets of features or instances. Figure 14B shows the feature importance of clinical data as per the Kernel SHAP. The bars against the features ‘Oral Contraception’ and ‘Family History’ are highly influential in deciding ‘Class 1 and 2’ when compared with ‘Class 0’.

From Figures 12–14, it can be concluded that ‘CEA’ and ‘CA19-9’ are the top two features highly contributing to model prediction. Furthermore, it can be noted that Kernel SHAP performs the best in comparison with Tree and Sampling SHAP, showing consistency in explaining model prediction across all three classes.

**Figure 15:**
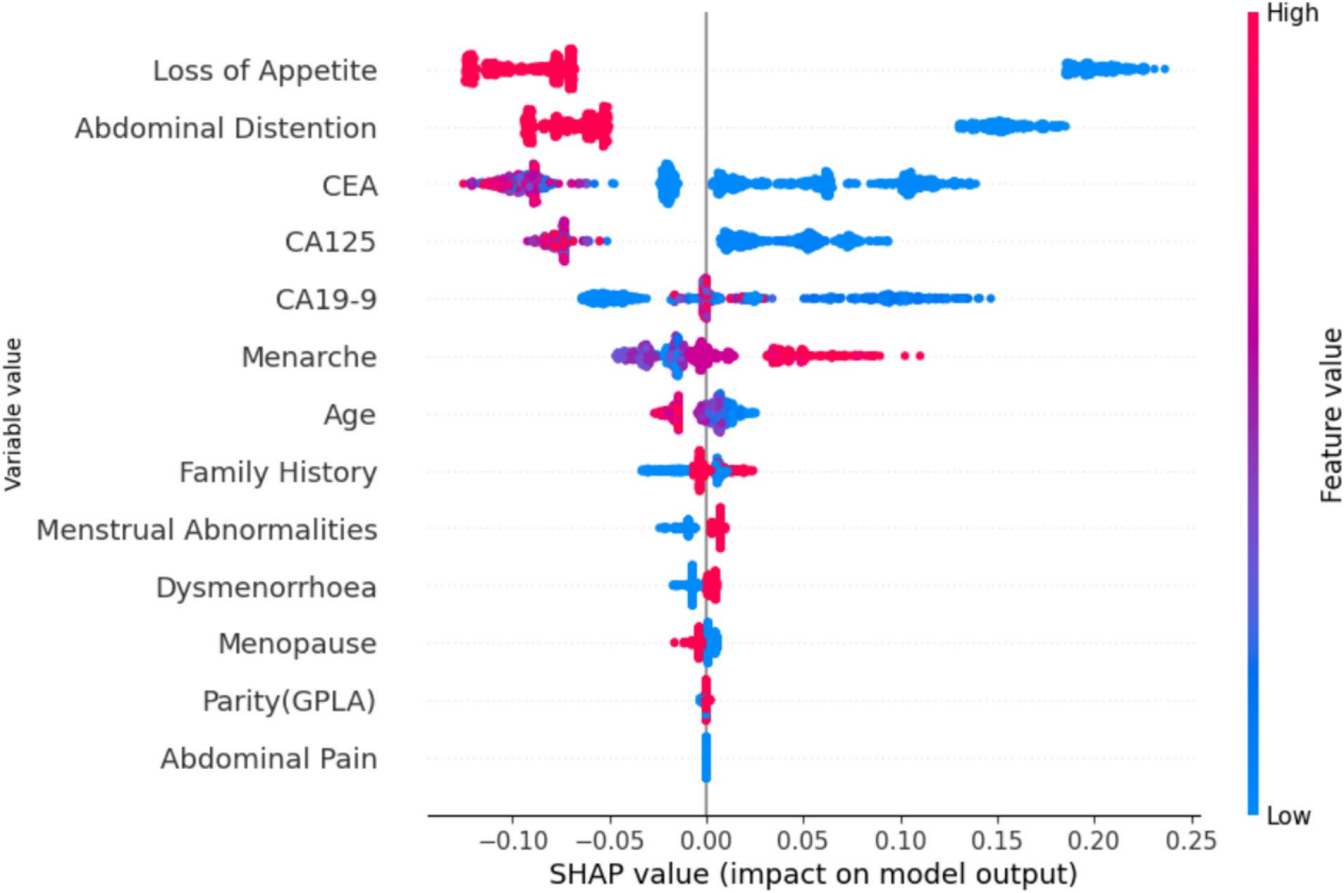
SHAP values based on the proposed model.

**Figure 16:**
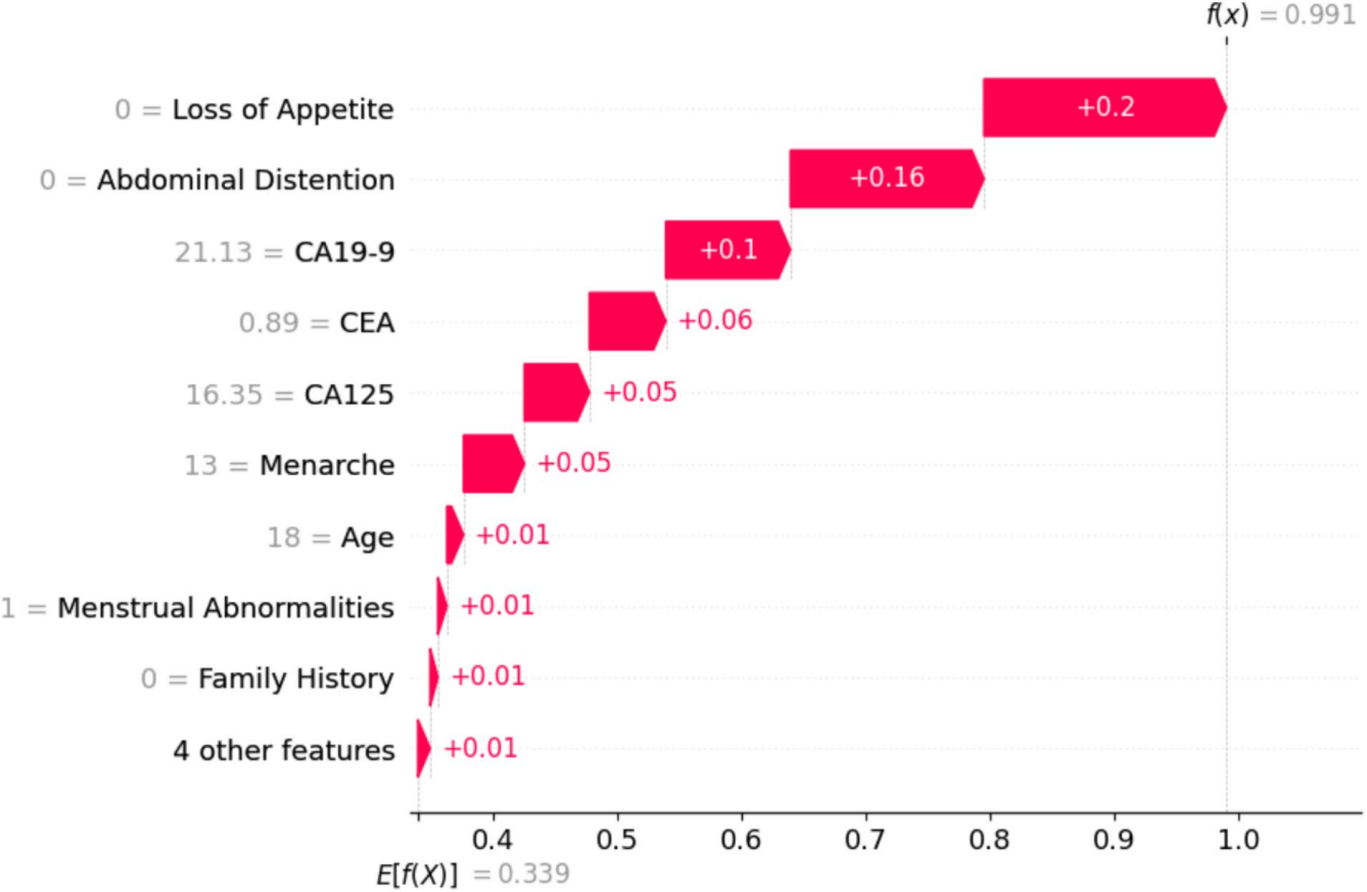
Training sample of a benign case with a probability of 99.1%.

Figure 15 shows the global view of the RF distribution, which corroborates the feature importance deduction that abdominal distention is one of the major contributing factors in classifying ovarian lesions. A mix of cases can be found in some instances that might not be a contributing factor but a few individual instances with relatively higher values could be studied further to understand the factors, if any, that set them apart. In conclusion, features with positive SHAP values for a particular instance can be indicative and helpful in stratifying a case. SHAP values will help in identifying the features that contribute to the model predictions. In Figure 16, the top nine features contributed to predicting particular training instances as benign cases. From the figure, it can be observed that while getting trained, the features have contributed to learning and the correct classification. It is also evident from the values that the features such as loss of appetite, abdominal distention, and family history are absent, the tumor markers such as CEA, CA19-19, and CA125 values are very much within the safe range, and the value of the most important feature Age is 18. This shows that SHAP has predicted the case as benign with a probability of 99.1%.

**Figure 17:**
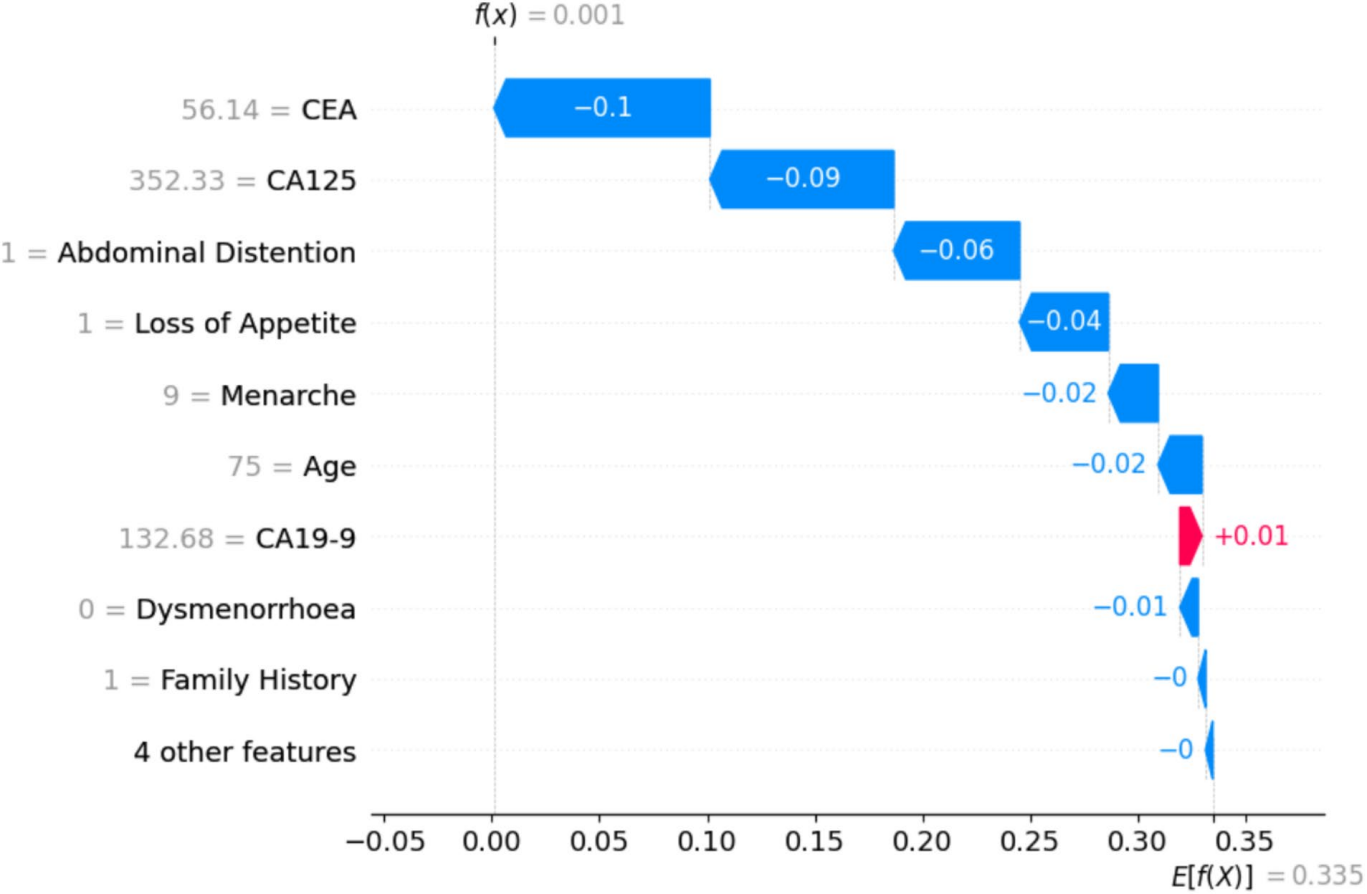
Training sample of a benign case with a probability of 0.1, which is a malignant case.

Figure 17 shows that the particular instance is classified as not non-benign, with a probability of 0.1. It is evident from the value on the left-hand side that the values of tumor biomarkers CEA, CA125, and CA19-9 are very high. The values of the clinical parameters such as family history, loss of appetite, and abdominal distention are all positive, with age being 75. From Figures 16, 17, it can be confirmed that the values of these features vary with huge differences.

*p*-value is the measure that determines the significance of the obtained results in the hypothesis tests. It represents the probability of obtaining the test results at least as extreme as the observed results. Cohen’s *d* value is often used to evaluate the practical significance of a model’s performance and the impact of the intervention. It can be observed from Table 1 that for CEA, loss of appetite, CA19-9, CA125, and abdominal distention, the p-value is extremely small (less than 0.5), indicating that the result is highly statistically significant. Cohen’s *d* values of these variables suggest a large effect size, which means the impact of these variables on diagnosis is very significant.

**Table 1:** *p*-value and effect size (Cohen’s *d*) for variables (influencing diagnosis and classification of ovarian mass lesions) ranked top 5 by SHAP.

Ranking	Variables	*p*-value	Cohen’s *d* value
1	CEA	3.7974 × 10^−10^	−1.4000
2	Loss of appetite	1.2299 × 10^−8^	3.2861
3	CA19-9	2.1076 × 10^−11^	−1.3615
4	CA125	0.1115	0.0883
5	Abdominal distention	1.2299 × 10^−9^	3.2861

From Table 2, it can be observed that *p*-values are significantly larger and Cohen’s d values are greater than 0.2, which indicates a much smaller effect size when compared with the most important variables. The negative Cohen’s d values suggest a negative effect.

**Table 2:** *p*-value and effect size (Cohen’s *d*) for variables (influencing diagnosis and classification of ovarian mass lesions) ranked bottom 5 by SHAP.

Ranking	Variables	*p*-value	Cohen’s *d* value
16	Menstrual abnormalities	4.1922	−0.4490
17	Dysmenorrhea	4.1922	−0.4490
18	Menopause	2.4639	0.4083
19	Parity (GPLA)	5.3066	−0.3210
20	Abdominal pain	1.0	0.0

Table 3 shows the difference in ranking based on SHAP values and p-values, which implies that while some features have more predictive capabilities, it might not be statistically as evident. Abdominal distention ranks higher in SHAP values than in p-values, indicating that it might be more important for prediction than its statistical significance might indicate.

**Table 3:** Comparison of rankings generated by SHAP values and *p*-values.

Rank	Feature	SHAP value rank	*p*-value rank
1	CEA	1	4
2	Loss of appetite	2	1
3	CA19-9	3	5
4	CA125	4	3
5	Abdominal distention	5	2
6	Family history	6	6
7	Menarche	7	8
8	Age	8	7
9	Menstrual abnormalities	9	9
10	Dysmenorrhea	10	10

## 5 Discussion

In this section, we compare the results of the proposed model with those of the existing studies mentioned in Section 2.

After a rigorous survey of the available literature, from Table 4, we found that there are not many studies in the literature that incorporate a combination of clinical, radiological, and serum-based parameters for ovarian cancer diagnosis and prognosis, which is an advancement in the state of the art beyond the existing literature—this mapping of multi-modal data onto a common subspace benefit from the complementary information in each modality. Therefore, we attempted to fill this gap in the literature, specifically patient data available in clinical data and radiological and tumor markers. Learning these data proceeds by maximizing intra-class similarities and inter-class differences to obtain richer representations. The richer representations obtained from these data trained ensemble-based classifiers to diagnose cancer. The proposed approach also aligns with medical experts using information from different sources to corroborate and conclude their diagnosis.

**Table 4:** Comparison with the existing relatable studies.

Literature	Dataset used	Type/modality of data	Number of samples considered	Number of features	No. of patients	Parameters used in the dataset	Methods used	Performance reported	XAI methods
Nopour et al. (5)	In-house	Clinical data	1,473	26	1,473	Age, Family history, family cancer syndrome, etc.	XGBoost, SVM, KNNs, ANN, RF	XGBoost: AUC–ROC = 0.93 (95% CI: [0.91–0.95])	Not used
Lavanya J M et al. (13)	Open access	Clinical data	349	49	349	22 general chemical tests, 19 blood routine tests, and 6 tumor markers, including age and menopause information	KNNs, SVM, Decision Trees, Max Voting, Boosting, Bagging, Stacking	Base accuracy = 85%, Stacking: 89%	SHAP
Abuzinadah et al. (22)	Open access	Clinical data	349	49	349	22 general chemical tests, 19 blood routine tests, and 6 tumor markers, including age and menopause information	Stacked Ensemble Model (Bagging + Boosting)	Accuracy = 96.87%	SHAP
Schilling et al. (10)	Open access	Gene expression	4,490	9,954	585	mRNA sequencing data	K-means, Naive Bayes, Logistic Regression, SVM	F1 score = 0.89	SHAP
Chao et al. (9)	In-house data	demographic and clinicopathologic features	6,809	67	6,809	Age, dysmenorrhea, Family history, CA125, Leiomyoma, etc.	Gradient-Boosting Decision Tree, Logistic Regression	LR: AUC = 0.891, Sensitivity = 88.9%, Specificity = 76.7%	Not used
Ahamad et al. (18)	Open access	Clinical data	349	49	349	22 general chemical tests, 19 blood routine tests, and 6 tumor markers, including age and menopause information	RF, SVM, DTs, XGBoost, LR, GBM, LGBM	Accuracy = 91%	Not used
Chen et al. (25)	Open access	Gene expression	4,490	9,954	585	mRNA sequencing data	Gradient Boosting Decision Tree	Accuracy = 96.5%	Not used
Zeng et al. (23)	Open access	Gene expression	4,490	9,954	585	mRNA sequencing data	Machine learning models (not specified explicitly)	AUC: 0.952 (BRCA1), 0.911 (multi-omics model)	Risk Score Interpretation for Survival Prediction
Proposed work	In-house	Clinical data, ultrasound parameters, and Tumor markers	1,500	20	1,500	CEA, CA19-9, CA125, Loss of Appetite, Abdominal Distention, Family History, Age of Menarche, Age, etc.	Three-stage ensemble model (SVM, RF, XGBoost)	Accuracy = 98.66%	LIME and SHAP

Hence, the innovative component and knowledge addition in this study is a unique combination of specific problem definition, usage of more relevant and near-match parameters that clinicians follow in their day-to-day practice, and translation of this cognition into an AI system, resulting in improved performance with reliability, consistency, and accuracy.

## 6 Conclusion and future studies

This study demonstrates the development and application of an ensemble classifier along with explainability to accurately predict benign, borderline, and malignant ovarian cancers using clinical, ultrasound, and tumor marker data. It has been proved experimentally that the proposed three-stage ensemble architecture exhibited higher performance than the existing models in the literature, with an accuracy of 98.66%.

As a future direction, it is envisioned that if the subcategories of malignant ovarian cancer and histopathological images are considered in the classification process, performance may be affected, leaving lacunae for further development in the future.

The developed classification paradigm in this study has the potential to bring translational changes in the ovarian cancer management protocols in clinics, in particular, which can help in lowering the cost of treatment, lowering morbidity, and reducing the risk of mortality specifically for women in resource-constrained and economically challenged scenarios.

## Data Availability

The raw data supporting the conclusions of this article will be made available by the authors, without undue reservation.
